# Development of a method for walking step observation based on large-scale GPS data

**DOI:** 10.1186/s12942-022-00312-5

**Published:** 2022-09-07

**Authors:** Shohei Nagata, Tomoki Nakaya, Tomoya Hanibuchi, Naoki Nakaya, Atsushi Hozawa

**Affiliations:** 1 Graduate School of Environmental Studies, Tohoku University, 468-1 Aoba, Aramaki, Aoba-ku, Sendai, 980-0845 Japan; 2 Department of Traffic and Medical Informatics in Disaster (Endowed Research Division), Tohoku Medical Megabank Organization, Tohoku University, 2-1 Seiryo-machi, Aoba-ku, Sendai, Miyagi 980-8573 Japan; 3 Graduate School of Medicine, Tohoku University, 2-1 Seiryo-machi, Aoba-ku, Sendai, Miyagi 980-8573 Japan

**Keywords:** Global positioning systems, Physical activity, Walking, Human mobility, Big data

## Abstract

**Background:**

Widespread use of smartphones has enabled the continuous monitoring of people’s movements and physical activity. Linking global positioning systems (GPS) data obtained via smartphone applications to physical activity data may allow for large-scale and retrospective evaluation of where and how much physical activity has increased or decreased due to environmental, social, or individual changes caused by policy interventions, disasters, and infectious disease outbreaks. However, little attention has been paid to the use of large-scale commercial GPS data for physical activity research due to limitations in data specifications, including limited personal attribute and physical activity information. Using GPS logs with step counts measured by a smartphone application, we developed a simple method for daily walking step estimation based on large-scale GPS data.

**Methods:**

The samples of this study were users whose GPS logs were obtained in Sendai City, Miyagi Prefecture, Japan, during October 2019 (37,460 users, 36,059,000 logs), and some logs included information on daily step counts (731 users, 450,307 logs). The relationship between land use exposure and daily step counts in the activity space was modeled using the small-scale GPS logs with daily step counts. Furthermore, we visualized the geographic distribution of estimated step counts using a large set of GPS logs with no step count information.

**Results:**

The estimated model showed positive relationships between visiting high-rise buildings, parks and public spaces, and railway areas and step counts, and negative relationships between low-rise buildings and factory areas and daily step counts. The estimated daily step counts tended to be higher in urban areas than in suburban areas. Decreased step counts were mitigated in areas close to train stations. In addition, a clear temporal drop in step counts was observed in the suburbs during heavy rainfall.

**Conclusions:**

The relationship between land use exposure and step counts observed in this study was consistent with previous findings, suggesting that the assessment of walking steps based on large-scale GPS logs is feasible. The methodology of this study can contribute to future policy interventions and public health measures by enabling the retrospective and large-scale observation of physical activity by walking.

## Background

Physical inability is a major risk factor for non-communicable diseases, and insufficiently active people have a 20–30% increased risk of death than sufficiently active people [1]. The prevalence of physical inactivity in high-income countries has increased since 2001 [2], and more than a quarter of the adult population worldwide is inactive [1]. The “Global Action Plan on Physical Activity 2018–2030: More Active People for a Healthier World,” published by the World Health Organization in 2018, calls for a 15% reduction in the prevalence of physical inactivity among adults and adolescents by 2030 [3]. The World Health Organization also suggested that policy actions for improving the social, cultural, economic, and environmental factors that support physical activity and individually focused approaches are required to achieve this goal [3]. Therefore, evaluating how environmental, social, and individual factors affect an increase or decrease in active mobility such as walking and cycling is crucial to the planning of feasible policy actions and interventions.

In recent years, many studies have effectively used global positioning systems (GPS) and accelerometers to objectively capture the relationship between the amount of physical activity and environmental exposures in people’s active spaces [4–8]. For instance, by utilizing these technologies, it was demonstrated that increased time spent in green spaces promoted vigorous-intensity physical activity in children [5] and light-intensity and moderate-to-vigorous-intensity physical activity in late middle-aged adults [7]. Rundle et al. [6] showed that people tended to visit walkable neighborhoods, and neighborhood walkability was associated with moderate-intensity physical activity. Furthermore, Jansen et al. [8] found that, although people tended to spend more time outside the neighborhood, effects of land use types—such as green space, blue space, and shops and foodservice places—on increased moderate-to-vigorous-intensity physical activity decayed with distance from home. The objective approaches demonstrated in these previous studies made it possible to assess to what degree physical activity volume can be increased or decreased by people’s exposure to environmental factors; however, GPS accelerometer-based investigations often require specialized mobile devices, thereby making extensive surveys costly.

Currently, the increased availability of human mobility data obtained by personal mobile devices or ubiquitous sensors incorporated into urban systems has led many researchers to attempt large-scale observations of people’s movements [9, 10]. However, the use of such data for physical activity measurement is still limited. Previous studies applied the large-scale mobility data collected from mobile phone users to clarify people’s daily travel regularity [11], characteristics of tourist behavior [9], differences in consumption behavior based on lifestyle [12], and the regularity and temporal trends of bicycle trips [13, 14]. As a rare example of health-related GPS-based research, Woodcock et al. [15] analyzed more than 550,000 users’ trips in one year and showed that the health benefits of an increase in physical activity by a bicycle sharing system in London outweighed the negative impact due to the occurrence of traffic accidents and exposure to air pollution; however, they did not directly measure users’ physical activity amounts.

Apart from the GPS issue, many smartphones also constantly monitor and record people’s physical activity; these data have been utilized for recent research. Tison et al. [16] showed that people’s step counts decreased by 27.3% worldwide within 30 days of the declaration of the coronavirus disease 2019 (COVID-19) pandemic, based on individual-level step count data obtained by a wellness smartphone application. Nagata et al. [17] also retrospectively observed decreased physical activity due to preventive measures against infections or decreased amount of work during the COVID-19 outbreak in Japan by using step count data recorded by the iPhone healthcare application.

In the current situation where people’s movements and physical activities are continuously monitored by smartphone applications and data are readily available, it should be possible to retrospectively assess the impact of policy interventions or unexpected events such as pandemics and natural disasters on physical activity based on changes in human mobility on a large scale by linking people’s movements and physical activities observed by these systems. However, several technical issues prevent the utilization of large-scale GPS data for physical activity measurement. First, although the amount of physical activity varies according to individual characteristics such as sex, age, and socioeconomic status in general [18], spatial big data, including commercial application-based GPS data, often do not have those attributes because they are collected not for scientific research but for specific commercial needs in many cases [19]. Second, because many GPS logs—the list of GPS measurement records with location information and time stamps—are stored and provided at non-uniform time intervals due to applications’ specifications and masking techniques for privacy protection [20], movement trajectories are often only incompletely understood. Third, as data with both GPS-based location logs and the amount of physical activity are still limited, it is difficult to link movement trajectories to physical activity by large-scale GPS data.

In Japan, large-scale GPS data collected by commercial applications are sold by several private companies. In addition, GPS data with daily step counts collected from a pedometer application are also available for purchase, although the data size is not as large. To implement a dynamic observation of people’s walking conditions within urban areas based on large-scale and incomplete GPS logs that hold only location information, this study attempted to develop a movement trajectory-based simple method for daily step count estimation.

## Methods

This section explains the dataset, processing steps, and method of statistical modeling for building the daily step count estimation model. For preparing the data, we used the following standardized grid squares defined as a unified parcel framework by the Japanese government [21]: (i) Basic Grid Square (hereinafter referred to as a 1 km Grid Square), represented by 30″ latitude × 45″ longitude (ca. 1 km × 1 km) grid squares; (ii) Half Grid Square (hereinafter referred to as a 500 m Grid Square), which is the Basic Grid Square divided equally into four parts (two by two); (iii) Quarter Grid Square (hereinafter referred to as a 250 m Grid Square), which is a Half Grid Square divided equally into four parts (two by two), and iv) 1/10 Grid Square (hereinafter referred to as a 100 m Grid Square), which is a Basic Grid Square divided equally into 100 parts (10 by 10). In Japan, this framework is used to aggregate statistical and geographic information in various situations such as national or local governments’ urban land use and disaster mitigation planning and private companies’ area marketing [21]. The code corresponding to the grid square can be calculated based on latitude and longitude [22].

### Data collection

#### GPS logs

For travel history data, we employed ‘dynamic population data’ that include GPS logs of people who use smartphone applications developed using a special software development kit provided by Agoop Corp. Although ordinary commercial GPS data do not include information on physical activity associated with users’ movement, a unique feature of the Agoop data is that some GPS logs obtained by a pedometer application called WalkCoin (Agoop Corp.) include the number of steps taken. We chose these data to link the GPS-based movement trajectories with step count, an indicator involving physical activity. The GPS logs were obtained not only when the application was running in the foreground but also in the background. The minimum time unit in the GPS logs was one minute.

We identified individuals whose location was obtained within Sendai City, the prefectural capital of Miyagi, Japan, between October 1 and 31, 2019, as the target users, and we were provided their GPS locations observed in October 2019. Sendai City is located approximately 300 km north of Tokyo and is the regional capital of Japan’s Tohoku (northeast) region with a population of approximately 1.1 million as of 2020 [23, 24]. It takes about one hour and 40 min from Sendai Station to Tokyo Station by the Tohoku Bullet Train [23]. There are subways connecting the city center to suburban areas of Sendai in the north–south and east–west directions, and according to a Sendai City report, as of 2021, 99.1% of the population lived within 1 km of a train station or 500 m from a bus stop [25]. In the suburbs, however, high automobile dependence is one of the issues to be solved [25]. Sendai City has set a goal of achieving a transportation system centered on public transportation and is trying to increase people’s mobility by creating walkable spaces and improving the convenience of public transportation and bicycles, especially in the city center [26].

**Fig. 1:**
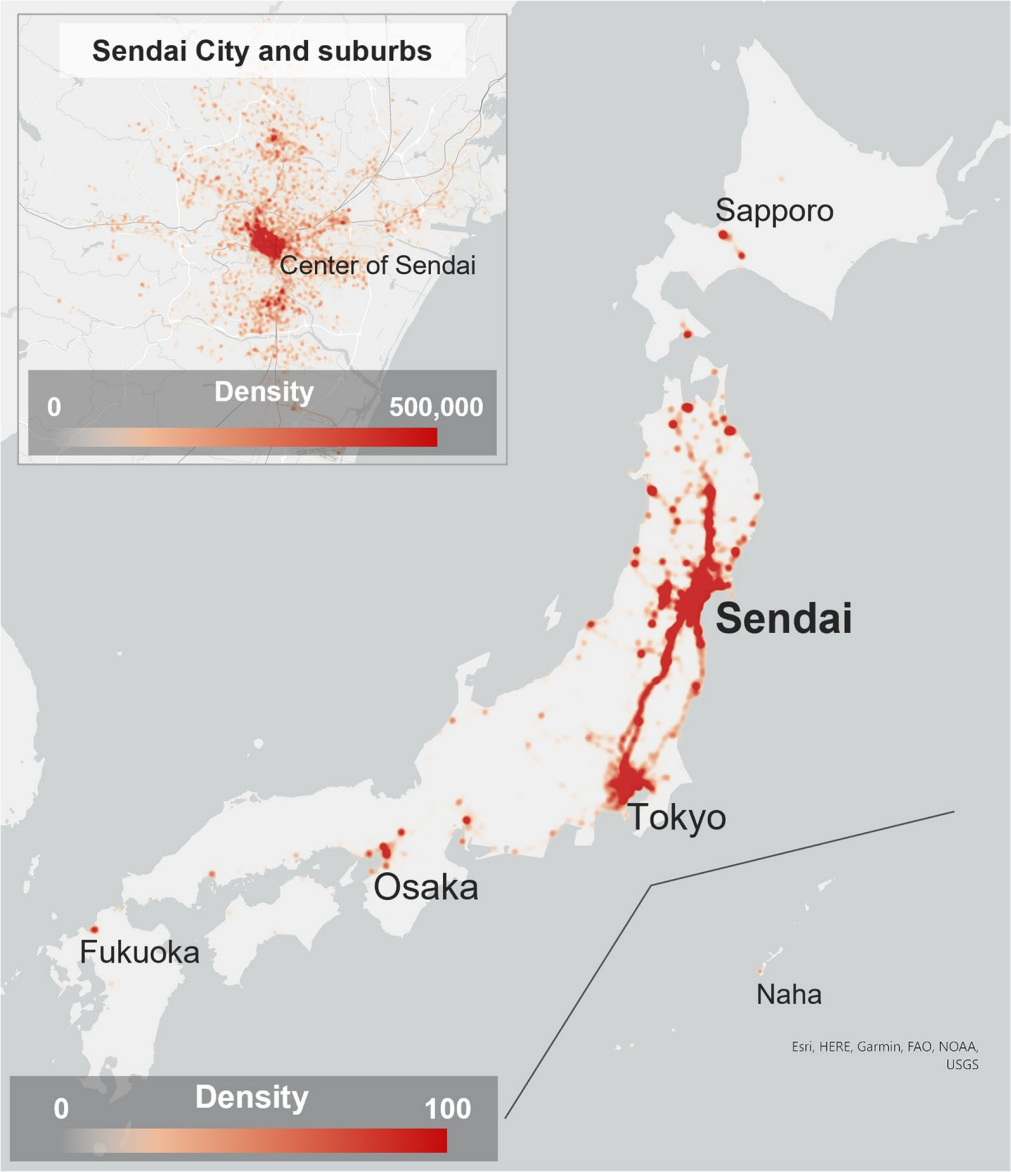
Density of GPS logs. Density (number of GPS logs per square kilometre) was clculated by the kernel density tool of ArcGIS Pro (Ver. 2.8.3). For the density of all of Japan, the cell size was 1 km, and the bandwidth was 10 km. For the density around Sendai, the cell size was 50 m, and the bandwidth was 250 m

Of the target users’ GPS logs, we used those with a horizontal GPS positioning error of less than 200 m with a valid universally unique identifier (UUID) (36,059,000 logs of 37,460 users). Of those data, the logs obtained from WalkCoin included daily step count information (450,307 logs of 731 users). All WalkCoin data were obtained from iPhone users. To access step count information measured through sensors built into iPhones and the Apple Watch, including a triaxial accelerometer, gyroscope, and GPS [27], WalkCoin uses Core Motion functions [28] and HealthKit functions [29] of the application programming interface provided by Apple Inc. For the sex and age group of each user, we referred to the attribute of the log with the oldest timestamp for each UUID. The numbers of target users by sex were as follows: 11,080 male users (29.6%), 5,135 female users (13.7%), and 21,245 users of unknown sex (56.7%). Of them, WalkCoin users were as follows: 440 male users (60.2%), 272 female users (37.2%), and 19 users of unknown sex (2.6%). Only WalkCoin users had the age attribute, and their composition at the time of the first logging was: 10–19 years: 42 users (5.7%), 20–29 years: 219 users (30.0%), 30–39 years: 207 users (28.3%), 40–49 years: 164 users (22.4%), 50–59 years: 76 users (10.4%), 60–69 years: 17 users (2.3%), 70 years and over: two users (0.3%), and unknown: four users (0.5%). While the GPS logs were generally concentrated in and around Sendai City, they were distributed throughout Japan (Fig. 1).

Although the GPS logs contained a variety of information such as language settings of each user, estimated travel speed, and city code of estimated residence and workplace locations, the attributes used were as follows: UUID, year, month, day, hour, minute, latitude, longitude, GPS accuracy, sex, and age group. In addition, daily step counts were included only for WalkCoin users’ logs. The data were collected only from users who authorized the collection and provision of information to third parties, and users could stop providing their location information at any time. Furthermore, to protect user privacy, Agoop did not provide logs inside the 100 m Grid Square containing the estimated location of the user’s residence.

#### Land use data

As previous studies using GPS have often showed the relationship between land use types and peoples’ physical activity [5, 7, 8], we used the frequency of visits to each land use type as an indicator of environmental exposure. For land use data, we employed ‘Land Use by Subdivision Grids in Urban Area as of 2016’ published by the Ministry of Land, Infrastructure, Transport and Tourism, Japan [30]. In the data, each 100 m Grid Square within the urban area is classified into one of 17 land uses (Table 1). We selected 1 km Grid Squares overlapping land area of Japan and created a list of 100 m Grid Squares (*n* = 38,724,900) within the 1 km Grid Squares.

**Table 1:** List of land use types

Original land use types	Reclassified land use types
High-rise buildings	High-rise buildings
Dense low-rise buildings	Dense low-rise buildings
Low-rise buildings	Low-rise buildings
Factories	Factories
Public facilities	Parks and public spaces
Parks and green spaces	
Roads	Roads
Railways	Railways
Forests	Other
Wastelands	
Vacant lands	
Rivers and lakes	
Sea	
Seashores	
Golf courses	
Rice fields	
Other fields	

**Fig. 2:**
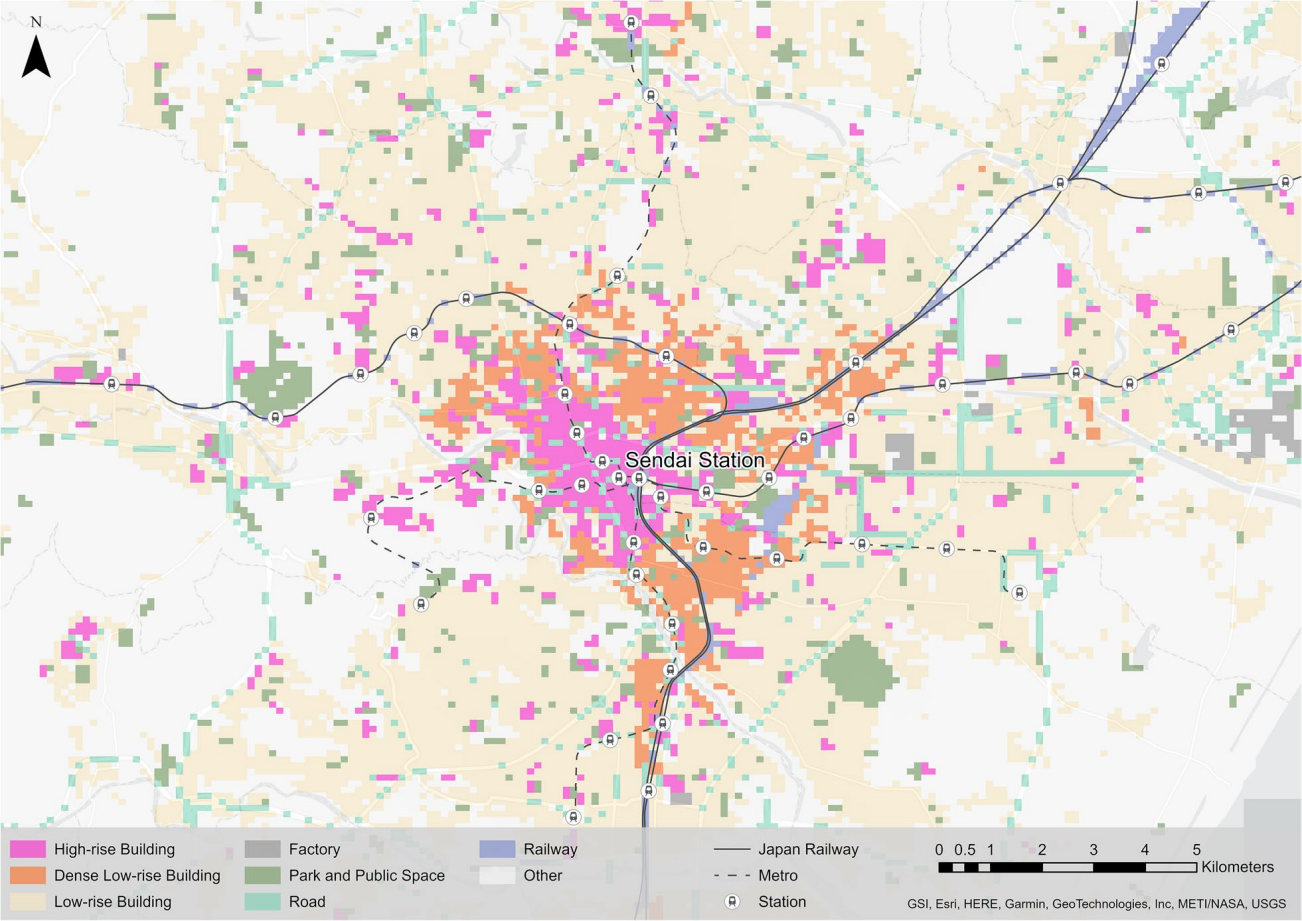
Spatial distribution of land use types in central Sendai City and the surrounding area

We reclassified 17 types of land uses into eight types as shown in Table 1. We used high-rise, dense low-rise, and low-rise buildings, factories, roads, and railways as defined originally, and defined ‘parks and public spaces’ by combining public facilities (e.g. public sports facilities) with parks (e.g. green spaces). The other non-urban land use types were defined as ‘other.’ In addition, grids with no land use information (i.e. those located outside the urban area) were classified as ‘other.’ Fig. 2 shows the distribution of land use in central Sendai and its vicinity.

### Linking GPS logs to land use data

**Fig. 3:**
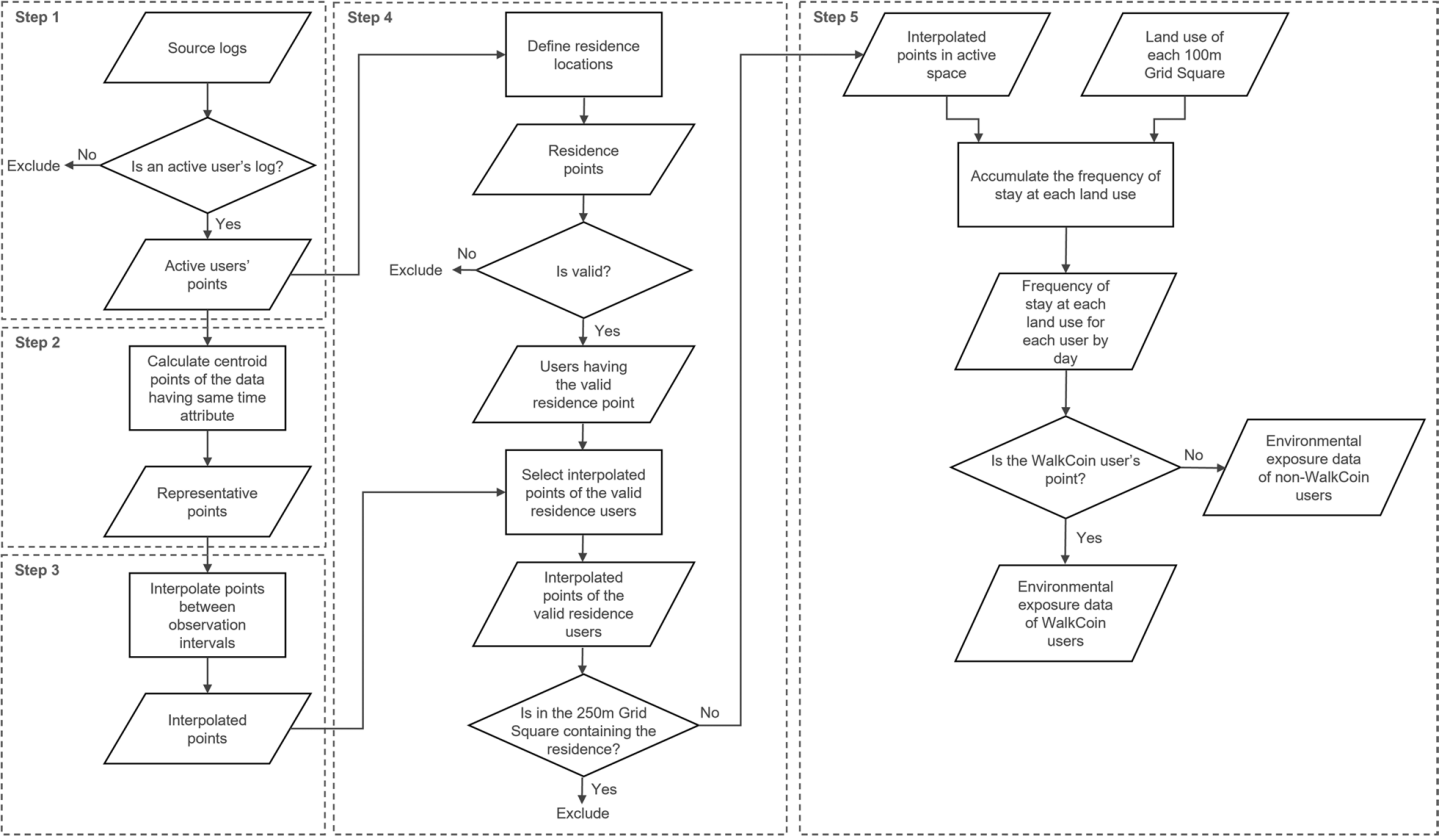
Processing flow for linking GPS logs to land use

The GPS logs and land use data were loaded into PostgreSQL (Ver. 14.1), a relational database management system. We used PostGIS extension (Ver. 3.2) to manage spatial database and geographic information system (GIS) processing. The location information of the data was stored as the geometry type referring to a geographic coordinate system (WGS 1984), and was cast to the geography type describing angular coordinates on a globe, for GIS processing. We also used Python (Ver. 3.9.9) to determine users’ residence locations. Figure 3 shows the data processing procedure for recovering movement trajectories based on the GPS logs that were incompletely recorded due to application specifications or privacy protection, and for linking the trajectories to land use.

#### Step 1: Selecting active users

Logs may have been insufficient to capture activity in situations where a user remained in the same location for a long period, did not carry their smartphone with the power turned on, or did not leave their residence. We assumed 12 h in a day as potential time for activities, and defined daily valid users as those who had 24 logs or more for each day, that is, users’ logs were observed at least once every 30 min during the potential time of activities. Moreover, because the observation of multi-day trip patterns was needed to estimate the users’ residences, users with more than 10 valid days were defined as active users and their logs were used in the next steps.

#### Step 2: Determining representative location of duplicate time logs

As the minimum time unit of the GPS logs was a minute, some location information showed time overlaps. To define a unique location corresponding to a certain time for a certain user, the central coordinates of the logs with the same UUID and time were calculated and employed as the representative locations.

#### Step 3: Interpolating points between observation points

**Fig. 4:**
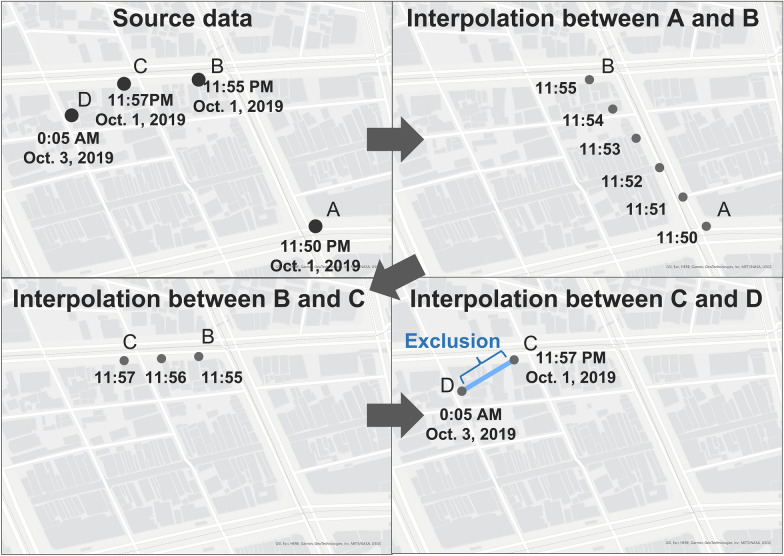
Method of the interpolation between observation points

This step reduced the incompleteness of movement trajectories due to the frequency of GPS logging. The GPS logs used were observed at irregular time intervals, and how users moved between two consecutive observations was unknown. Previous studies have proposed map matching methods to estimate unknown trajectories on roads due to the accuracy of GPS logs, based on the geometric or topological similarity with the road network and the hidden Markov models [31]. Rupi et al. [32] showed that cyclists’ GPS traces matched to road networks dedicated to bicycle travel correlated highly with the cyclists’ flows based on manual surveys. However, the map matching process often requires large-scale computational resources [33]. In this study situation, the GPS logs were distributed over a vast area of Japan, and it was sufficient to link the users’ movement to land use at a rough resolution of approximately 100 m rather than to a specific road. Therefore, we employed a simple interpolation method based on the position and time difference between observation points. Specifically, interpolation positions were defined as equally spaced intervals on a straight line between two consecutive logs in time, depending on the time difference. In case of an example shown in Fig. 4, four equally spaced points were created between A (observed on October 1, 2019 at 11:50 PM) as the starting point and B (observed on October 1, 2019 at 11:55 PM) as the ending point, and the interpolation times are stored as 11:51, 11:52, 11:53, 11:54 in order of proximity from A. Furthermore, in the example between B and C, the interpolation point was created at the midpoint because the time difference is only two minutes. In this step, the ST_LineInterpolatePoint function of PostGIS was used to create the interpolation points. As this function does not support the geography type-based angular distance measurement, interpolation position was calculated by the geometry type-based linear distance measurement.

In addition, no valid log was observed for October 2, 2019, between C (October 1, 11:57 PM) and D (October 3, 0:05 AM). We created interpolation points between C and D, and deleted the interpolation points for the invalid days with no logs in the representative points in Fig. 3. This step created the 284,811,885 interpolated points of 8555 users including 6,091,601 points of 221 WalkCoin users.

#### Step 4: Estimating the users’ residence locations

**Fig. 5:**
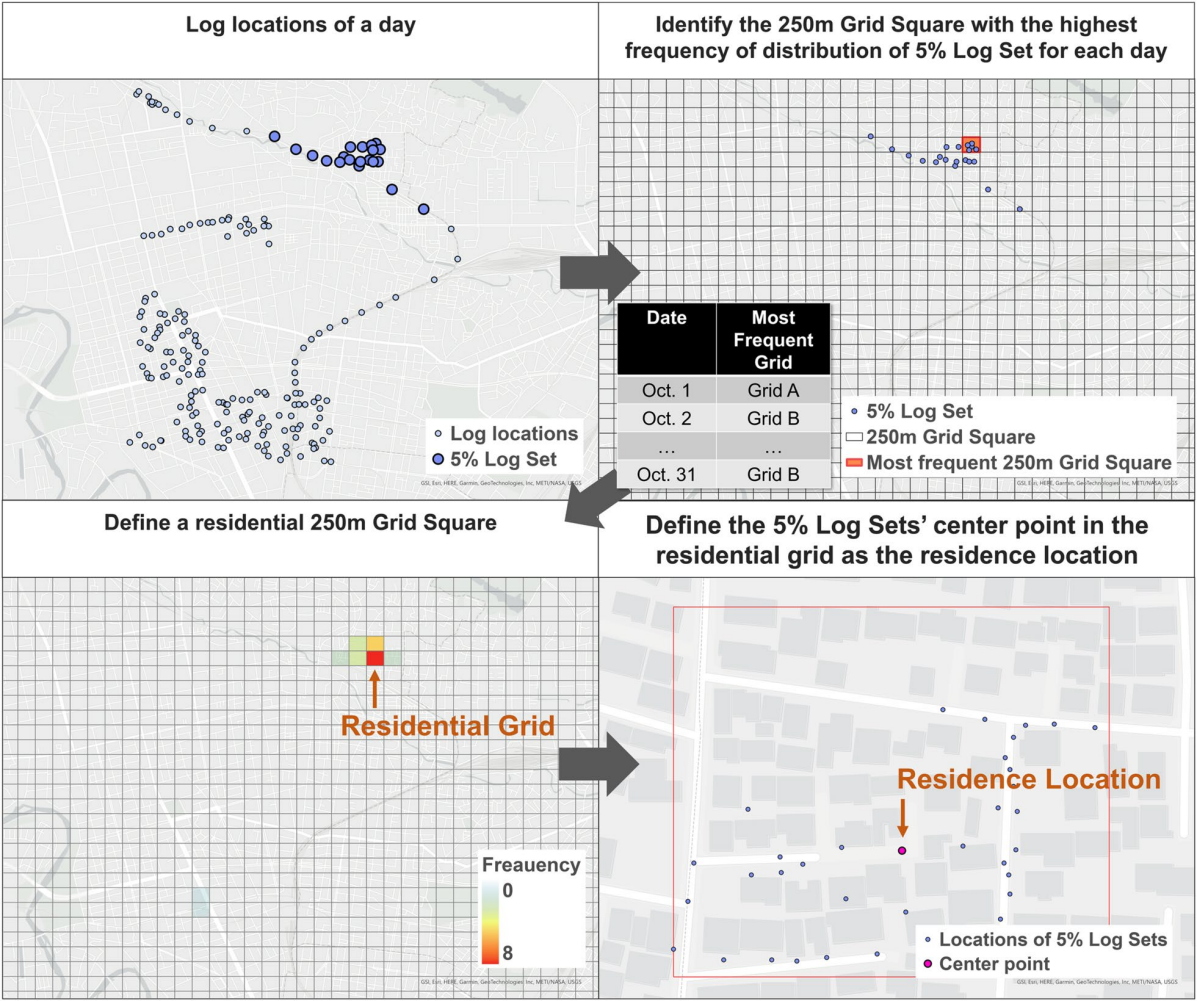
Method of residence location estimation. The 5% Log Set indicates the first and last 5% of each date’s logs for each UUID

We were not provided the GPS logs around users’ estimated residence. However, the environmental exposures around the users’ residence not involved in the activity may have been overestimated as the interpolated points between the end of one day and the first log of the next day created in Step 3. Therefore, we estimated the residential locations from the start and end locations of the daily logs, and removed the interpolated logs around there. Figure 5 shows the specific procedure for estimating the residence. First, the first and last 5% of each date’s logs (5% Log Set) for each UUID were selected from the active users’ points shown in Fig. 3. Second, the 250 m Grid Square with the most locations of 5% Log Set observed was identified for each combination of UUID and date. That is, if there were 31 days of valid logs for a given UUID, a list of the 31 most frequent grids by day was created. Third, the most-frequent grid in the list for each UUID was defined as the user’s residential grid. However, multiple most-frequent grids adjacent to each other or sharing the same adjacent grid were considered residential grids. If multiple most-frequent grids of a user did not all meet the above conditions—that is, if all the potential residential grids were located at a distance from each other—the user was excluded from the processing as residence undefinable. Finally, for each user, we defined the centroid location of the 5% Log Sets contained in the residential grid(s) as the residence location, and determined the valid users whose residence was located in Miyagi Prefecture. The Python library jismesh, available at PyPI, was used to determine the grid adjacencies.

Furthermore, we selected the points of the users with valid residence locations from the interpolated points, and excluded the points located in the user’s residential 250 m Grid Square. The output of this step, interpolated points of the valid residence users (Fig. 3), had 150,701,977 points of 8094 users including 4,631,808 points of 200 WalkCoin users.

#### Step 5: Linking the land use to the interpolated points

The frequency of each land use type intersecting with valid interpolated points outputted in Step 4 was used as the indicator of users’ daily environmental exposure. Considering the GPS positioning error, we summed up the land use types of 100 m Grid Squares whose centroid point was contained in the 100 m buffer zone from each interpolated point. The output of this process was stored by combinations of UUID and date.

In addition, we divided the output into data with daily steps (i.e. WalkCoin user-based data) used for building the model (environmental exposure data of WalkCoin users, Fig. 3) and data without steps used for estimating the number of daily steps (environmental exposure data of non-WalkCoin users, Fig. 3). We excluded the data with missing sex or age from WalkCoin user-based data. As for the final data indicating the daily environmental exposure used in the analysis, the WalkCoin user-based data had 3937 records of 198 users and the non-WalkCoin user-based data had 182,001 records of 7900 users.

### Model estimation

We built the model, which estimates daily step counts based on the frequency of exposure to each land use type, using the generalized additive model (GAM). Given the smartphone-carrying habits of each user, the model included user-specific random intercepts for adapting to the repeated observations of daily step counts by the same person during the study period. The model equation representing the relationship between daily totals of environmental exposures and daily step counts is as follows:

$${y}_{u,d}={\beta }_{0,u}+{\beta }_{1}{SEX}_{u,d}+{{\beta }_{2}AGE}_{u,d}+\sum_{k=1}^{K}{s}_{k}\left({x}_{u,d,k}\right)+{\varepsilon }_{u,d}$$

$${\varepsilon }_{u,d}\sim N(0,{\sigma }_{L1}^{2})$$

$${\beta }_{0,u}\sim N\left(\alpha , {\sigma }_{L2}^{2}\right)$$

$${x}_{u,d,k}=\mathrm{ln}\left({LANDUSE}_{u,d,k}+1\right)$$

where ${y}_{u,d}$ is daily step counts of user $u$, day $d$, ${x}_{u,d,k}$ is the environmental exposure index at land use type $k$, day d and ${\varepsilon }_{u,d}$ is an i.i.d. error. The environmental exposure ${x}_{u,d,k}$ is expressed as the logarithm of the value obtained by adding 1 to the frequency of each user’s visits to each land use type (high-rise, dense low-rise, and low-rise buildings, factories, parks and public spaces, roads, railways, and others) on each day, as summed up in Step 5 of the above procedure. $SEX$ is a variable that represents the sex of the user and takes binary values (1 for female and 0 for male users). $AGE$ is assigned the following values according to the user’s age group: 10: 10–19 years, 20: 20–29 years, 30: 30–39 years, 40: 40–49 years, 50: 50–59 years, and 60: 60–69 years. The data of users aged 70 years and over were removed in a series of pre-processing steps. ${\beta }_{0,u}$ is the user-specific random intercept, and ${\beta }_{1}$ and ${\beta }_{2}$ are the coefficients of $SEX$ and $AGE$, respectively. ${s}_{k}$ is a nonlinear function using smoothing splines corresponding to land use type $k$ [34]. We used R (Ver. 4.1.2) with mgcv package (Ver. 1.8.38) to estimate the model and determined that the relationship of the daily total exposures to daily steps was statistically significant if the *p*-value was less than 0.05.

### Mapping the daily step count distribution

We applied the fixed effects part of the fitted model to the environmental exposure data of non-WalkCoin users to obtain their expected daily step count distribution. As most users’ sex and age group was unknown for GPS data without step counts due to the specifications of the source applications, and no association was found between these variables and daily step count by the estimated model described below, the effects of sex and age group were ignored for the expected step counts of non-WalkCoin users.

After the daily step count estimation, we calculated mean daily steps for a weekday and weekend and holiday of each user. Additionally, to observe the impact of natural disasters on walking, we calculated the mean number of daily steps per weekend (i.e. October 5–6, October 12–13, October 19–20, and October 26–27, 2019). There were torrential rain disasters over a wide area of Japan on October 12–13, 2019, caused by Typhoon Hagibis, which had record intensity [35]. The Japan Meteorological Agency issued an emergency warning for Miyagi Prefecture on October 12 [36], and damages from landslides and flooding were reported in Sendai City [37].

To map the spatial distribution of daily steps, we calculated the mean of users’ mean daily step counts calculated by the above process, per 500 m Grid Square, based on the users’ residence locations. The geographic scope of the mapping was the 500 m Grid Squares within Sendai City, and grids with fewer than five residential users were excluded as non-assessable.

## Results

Table 2 shows the mean number of daily steps taken by sex and age group for the environmental exposure data of WalkCoin users (Fig. 3) used to estimate the model. In addition, Table 3 indicates the mean daily step counts according to the results of a pedometer-based survey conducted by the Miyagi Prefectural Government in 2016 [38]. Overall, WalkCoin users took more steps than the Miyagi Prefectural survey results. As for sex and age group, mean daily step counts of female users aged 20–29, 30–39, and 60–69 years and male users aged 40–49, 50–59, and 60–69 years were higher than the results of the Miyagi Prefectural survey.

**Table 2:** Mean number of daily mean step counts by sex and age group of WalkCoin users used for model estimation

	Total		Male		Female	
	n	Mean step counts	n	Mean step counts	n	Mean step counts
All age groups	198 (100%)	7710.72 (SD = 4979.94)	115 (100%)	7909.47 (SD = 4746.01)	83 (100%)	7389.56 (SD = 5322.29)
10–19 years	16 (8.1%)	9876.75 (SD = 4803.23)	7 (6.1%)	11,138.66 (SD = 4840.38)	9 (10.8%)	8522.91 (SD = 4390.37)
20–29 years	56 (28.3%)	8154.69 (SD = 5414.08)	27 (23.5%)	7900.91 (SD = 4365.41)	29 (34.9%)	8409.40 (SD = 6287.27)
30–39 years	53 (26.8%)	6908.63 (SD = 4979.90)	24 (20.9%)	6975.62 (SD = 5146.46)	29 (34.9%)	6841.75 (SD = 4811.77)
40–49 years	47 (23.7%)	7321.05 (SD = 4443.95)	39 (33.9%)	7571.46 (SD = 4574.99)	8 (9.6%)	6017.93 (SD = 3414.84)
50–59 years	21 (10.6%)	7635.19 (SD = 4492.40)	16 (13.9%)	8274.76 (SD = 4458.47)	5 (6%)	4560.35 (SD = 3217.03)
60–69 years	5 (2.5%)	8667.80 (SD = 4800.41)	2 (1.7%)	10,135.60 (SD = 3126.12)	3 (3.6%)	7200.00 (SD = 5693.31)

**Table 3:** Mean number of daily mean step counts by sex and age groups, according to a survey by the Miyagi Prefectural Government

	Total		Male		Female	
	n	Mean step counts	n	Mean step counts	n	Mean step counts
All age groups	613 (100%)	6024 (SD = 3512)	286 (100%)	6375 (SD = 3689)	327 (100%)	5716 (SD = 3320)
15–19 years	18 (2.9%)	6458 (SD = 3048)	13 (4.5%)	5401 (SD = 1728)	5 (1.5%)	9206 (SD = 3903)
20–29 years	31 (5.1%)	7855 (SD = 3895)	17 (5.9%)	8061 (SD = 4093)	14 (4.3%)	7604 (SD = 3624)
30–39 years	67 (10.9%)	6994 (SD = 3927)	30 (10.5%)	8202 (SD = 4113)	37 (11.3%)	6014 (SD = 3472)
40–49 years	108 (17.6%)	6990 (SD = 3523)	48 (16.8%)	7267 (SD = 3434)	60 (18.3%)	6769 (SD = 3576)
50–59 years	77 (12.6%)	6129 (SD = 3147)	34 (11.9%)	5937 (SD = 2696)	43 (13.1%)	6280 (SD = 3455)
60–69 years	162 (26.4%)	5723 (SD = 3190)	74 (25.9%)	5816 (SD = 3565)	88 (26.9%)	5644 (SD = 2835)
70 years and over	150 (24.5%)	4736 (SD = 3271)	70 (24.5%)	5557 (SD = 3841)	80 (24.5%)	4017 (SD = 2459)

Source: 2016 Miyagi Prefectural Health and Nutrition Survey (https://www.pref.miyagi.jp/soshiki/kensui/houkokusho28.html). SD, standard deviation.

**Fig. 6:**
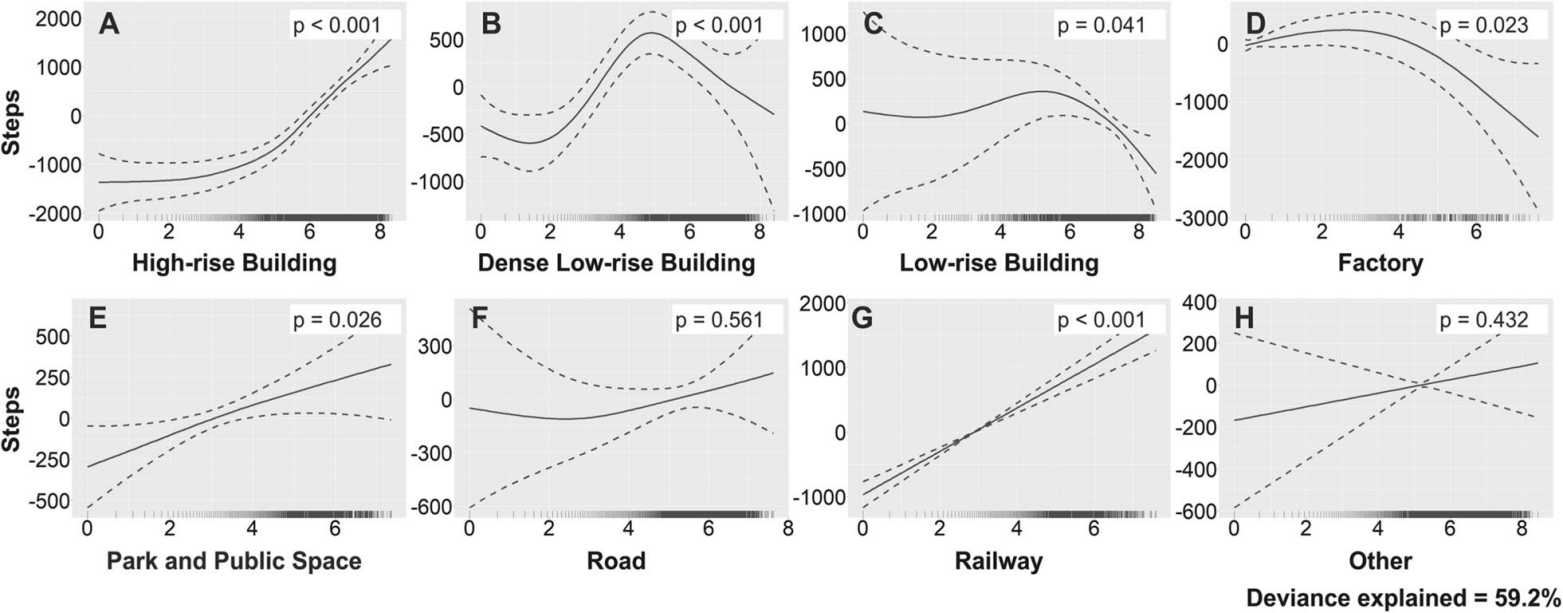
Relationships between steps and land use types estimated by GAM. The Y-axis of each graph is the number of steps and the X-axis is the logarithm of the value obtained by adding 1 to the frequency of visits to each land use type. Dashed lines indicate 95% confidence intervals

Figure 6 shows the relationship between daily step counts and the land use exposure index estimated by GAM (deviance explained = 59.2%). Judging from the *p*-values, statistically significant relationships between visits to high-rise (*p* < 0.001), dense low-rise (*p* < 0.001), and low-rise buildings (*p* = 0.041), factories (*p* = 0.023), parks and public spaces (*p* = 0.026), and railways (*p* < 0.001) and daily step counts were observed. As for the land use type positively related to daily step counts, if the frequency of visits to a high-rise building area exceeds a certain threshold, users are more likely to show increased daily step counts (Fig. 6A). Furthermore, the frequencies of visits to parks and public spaces and railway areas are linearly related to daily step counts (Fig. 6E, G). In contrast, if the frequencies of visits to low-rise buildings and factory areas exceed a certain threshold, they are negatively related to daily step counts (Fig. 6C). The relationship between the frequency of visits to dense low-rise buildings and daily step counts shows both positive and negative aspects depending on its frequency (Fig. 6B). No significant relationship between road and other areas and daily step counts was observed (Fig. 6F, H). In addition, sex and age group were not significantly related to daily step counts (Table 4).

**Table 4:** Estimated $\beta$ for sex and age

	$\beta$	95% CI	p
SEX	− 867.50	− 1913.51 to 178.52	0.104
AGE	− 19.67	− 61.90 to 22.57	0.361

*CI* confidence interval

**Fig. 7:**
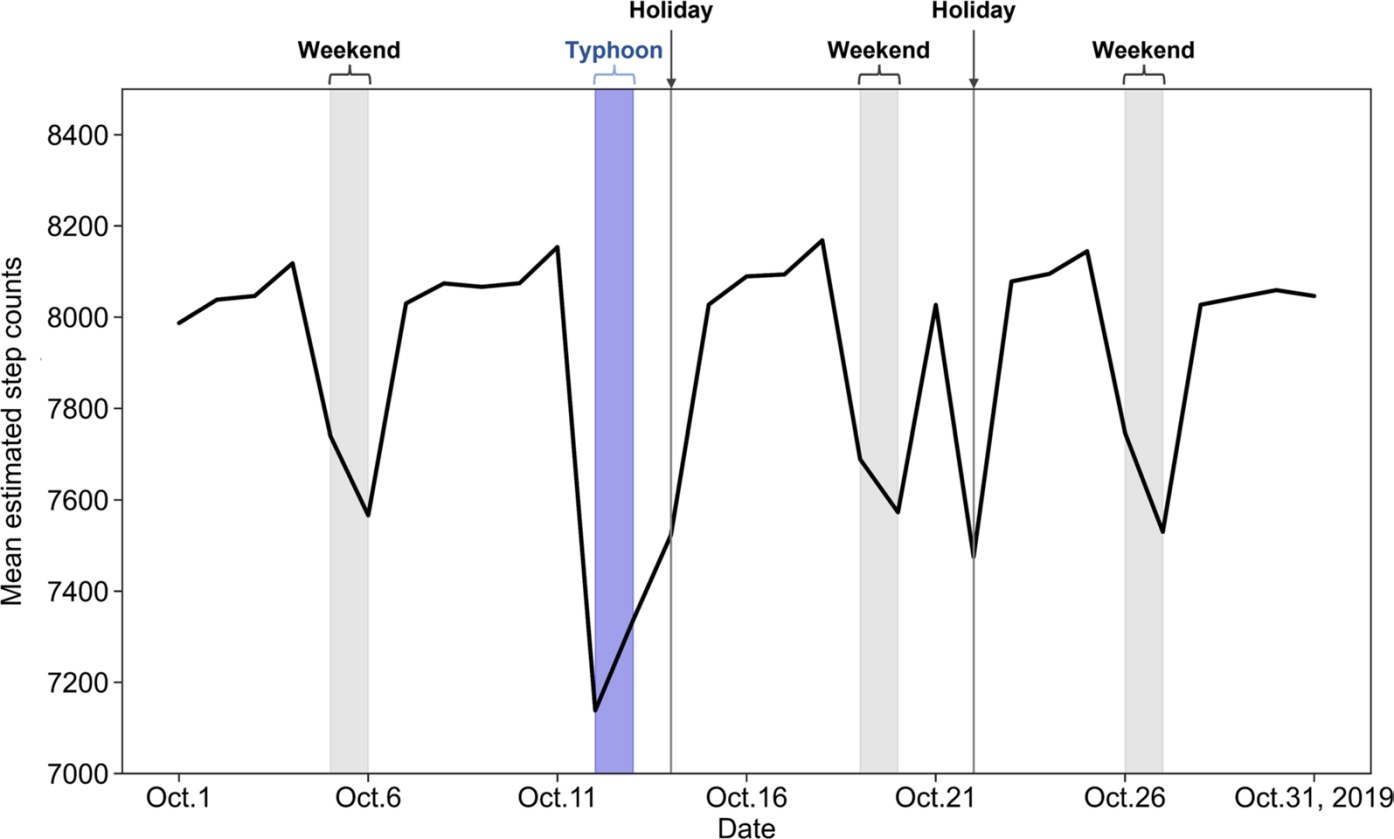
Changes in mean estimated step counts

**Fig. 8:**
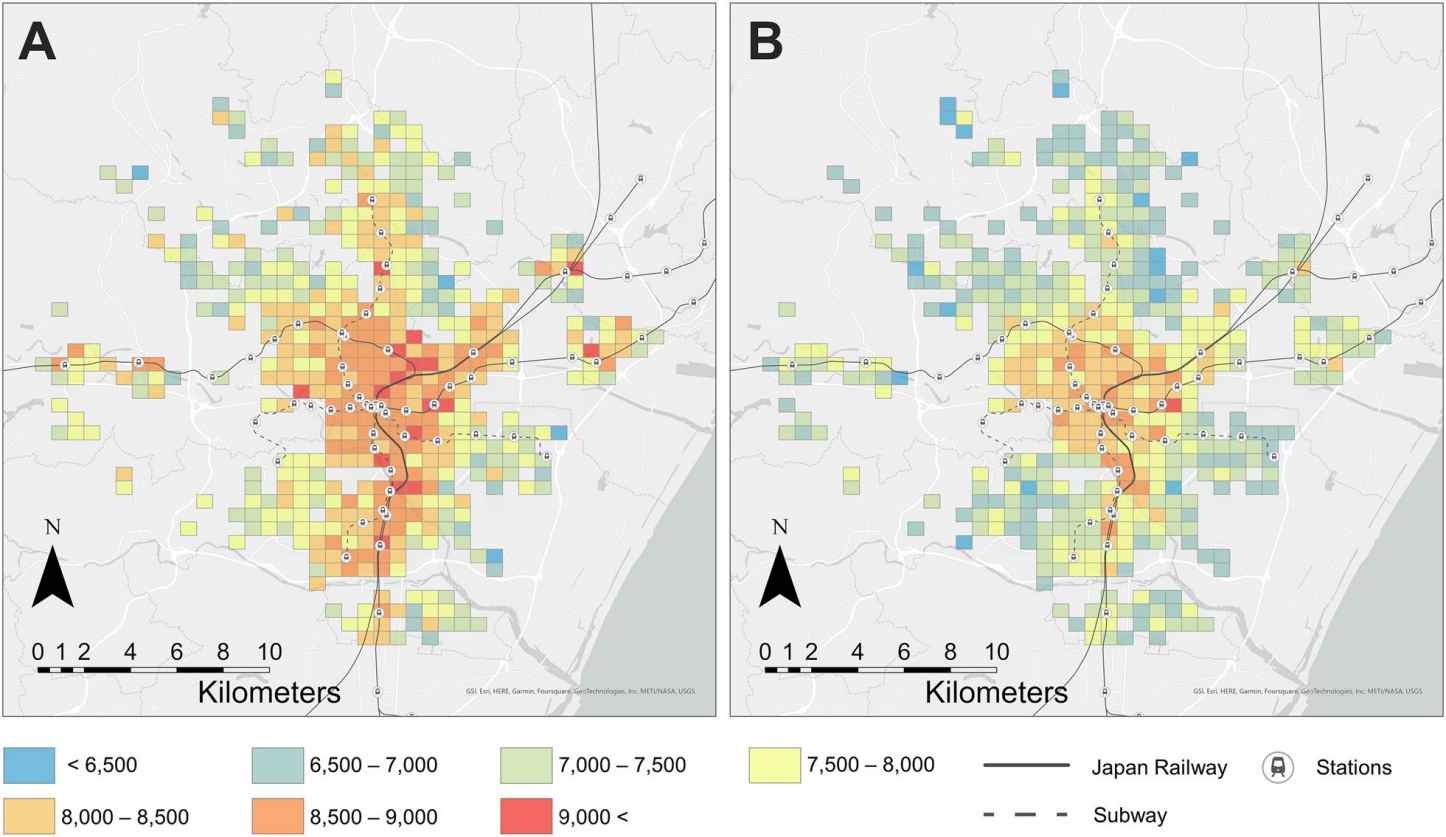
Spatial distribution of estimated step counts on weekdays (**A**) and weekends and holidays (**B**)

**Fig. 9:**
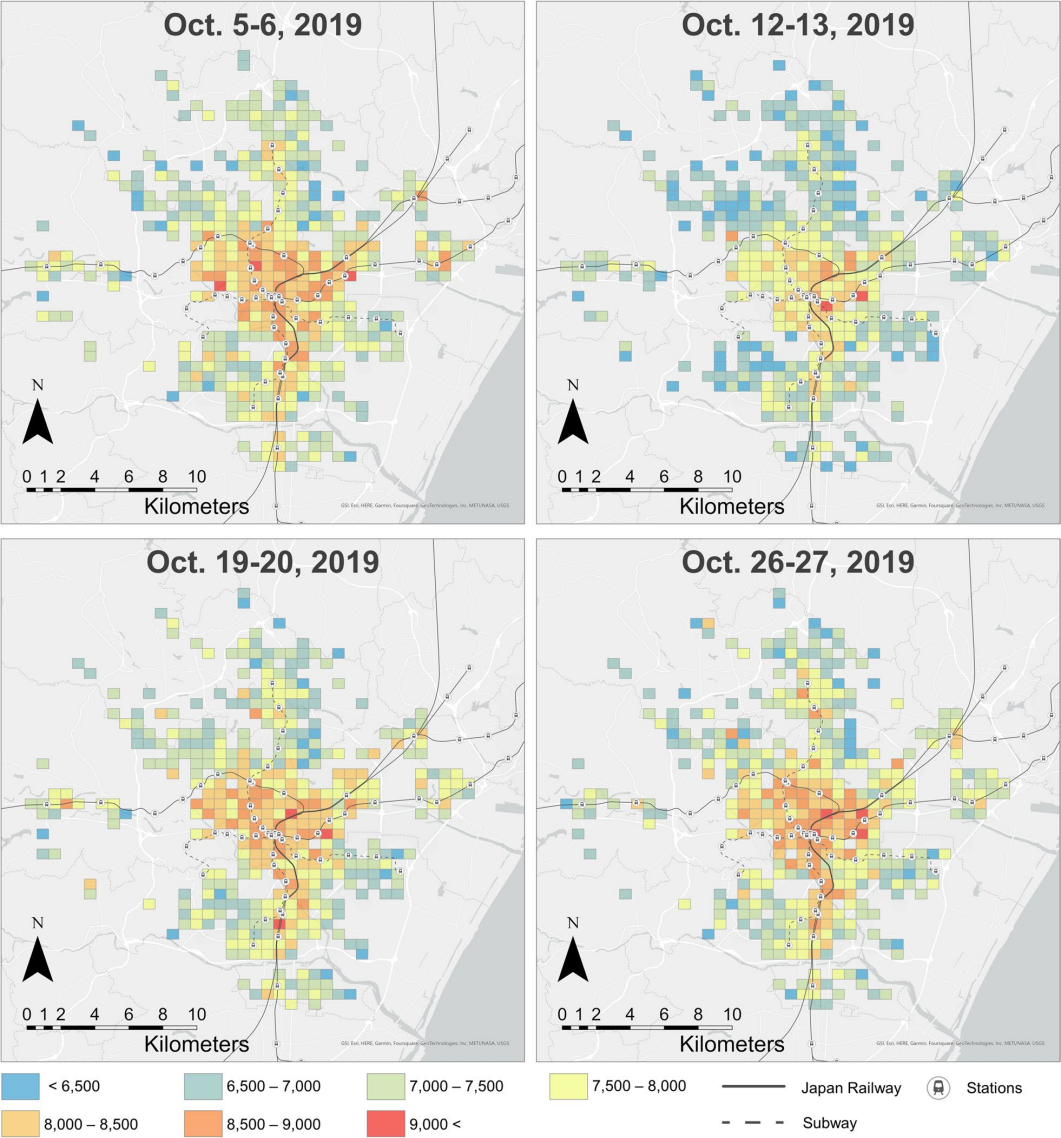
Spatial distribution of estimated step counts on each weekend

With regard to daily step count estimation based on the environmental exposure data of non-WalkCoin users (Fig. 3), the mean daily step count of prediction results was 7915.88 (standard deviation = 1570.50), approximately 1900 steps more than the daily step count from the Miyagi Prefectural Government survey results (6024, standard deviation = 3512), shown in Table 3. Figure 7 shows daily changes in the mean estimated step counts. Although the number of estimated steps tended to decrease on weekends and holidays, a clear decrease in steps was estimated on October 12–13, when the typhoon brought heavy rains. Figure 8 shows the spatial distribution of estimated daily steps (A: weekday, B: weekend and holiday). Overall, the weekday distribution shows a higher number of daily steps than the weekend and holiday distributions. Although residents of grids closer to the center of Sendai City tend to walk more on both weekdays and holidays, there are high step count areas around suburban railway or subway stations with the exception of the eastern area along the subway line. In a weekend comparison (Fig. 9), daily step counts on October 12–13, when the typhoon brought heavy rains, are clearly lower in the suburbs and areas away from the stations.

## Discussion

The widespread use of smartphones has enabled the dynamic and continuous monitoring of people’s movement and physical activity [10, 16, 17]. To implement the dynamic and retrospective observation of changes in the amount of physical activity, this study attempted to develop a simple method for people’s daily step count estimation based on large-scale GPS trajectories.

The number of mean daily steps of the WalkCoin users used for model estimation was higher than that reported in the Miyagi Prefectural survey [38], as shown in Table 3. This is expected, given that people who use pedometer applications are likely to actively walk, and logs of users staying around their homes were excluded in the data processing steps.

The model built using GAM showed the positive relationship of high-rise buildings, parks and public spaces, and railway areas with the daily step counts. The result for the high-rise building areas indicated that the daily step counts increase with longer stays or frequent visits to this area, rather than with short trips through there. In the study region, the majority of the high-rise building areas overlap with the commercial and business district in the center of Sendai City, which is high-density and has abundant destinations for shopping and work. In addition, there are many pedestrian-only paths in the area. Previous studies have consistently shown that environmental factors such as accessibility of destinations and the presence of pedestrian infrastructure are related to the amount of walking [39], and the results of this study also suggest that a highly accessible and walkable environment in the center of Sendai City promotes people’s active mobility, including walking.

In addition, the positive relationship between visits to parks and public spaces and daily step counts can support previous findings that the accessibility of parks, green spaces, and sports facilities is related to leisure-time and moderate-to-vigorous-intensity physical activity [7, 40]. The results of this study also suggest that parks and public spaces function as places to conduct activities such as walking, jogging, and dog walking.

The positive relationship between visits to the railway area and daily step counts can be explained in two contexts. The first is the possibility that the frequency of visits to the railway area reflects the implementation of car-independent active mobility. Many previous studies have shown that using public transportation promotes increased physical activity, as walking is required between home or the workplace and bus stops or train transit stations [41, 42]. However, the railway area itself may be a place where destinations are concentrated, as with the high-rise building area. If railway area exposure reflects only public transportation use, it is important to determine whether the railway was used during the day, and the linear relationship between increased exposure (i.e. increased train ride time) and increased daily step counts would not be expected, as the model suggests. That is, the increase in time spent at stations contained in the railway area may be driving the increase in step counts. As commercial facilities are generally concentrated in and around train stations in Japan, the results reflect increased step counts due to side trips around train stations, which can occur when using public transportation.

Furthermore, we observed a negative relationship between low-rise buildings and factory areas and daily step counts. Most of these areas are located in suburban areas, having an environment of low density and limited land use variations, suggesting increased car-dependent travel and reduced walking opportunities for users who frequently stay in these areas. In fact, a previous study in Japan demonstrated that the frequency of car use tended to increase among those who moved to low-rise residential areas and suburban areas [43].

In terms of the dense low-rise building areas, they are distributed between low-rise and high-rise building areas (Fig. 2), suggesting a mix of suburban residential and commercial areas. Therefore, the model estimation results express the effect of increasing step counts in high-rise building areas and the effect of decreasing step counts in low-rise building areas.

In the distribution of the estimated daily step counts based on the users’ estimated residence locations, although the residents’ daily step counts were higher in the center of Sendai City than in the suburbs on both weekdays and weekends and holidays, the decrease in step counts tended to be mitigated in the vicinity of the railway stations. The neighborhood walkability is often represented by a composite of factors such as intersection density and land use diversity related to accessibility around the residence [39, 44]. However, even if the residential neighborhood is not walkable, the high availability of public transportation promotes walking-based activities and daily active mobility in the urban area. Meanwhile, in the eastern areas along the subway line, there were areas where residents walk less, despite being in the vicinity of subway stations. This may be because the GPS logs were acquired less than four years after the opening of the subway line, and the lifestyle of the residents along the line was still car-dependent. Although the time period of the data used was limited, because the applications continually acquire data, it would be possible to apply this approach to observe how neighborhood environment changes due to opening of public transportation, urban redevelopment, and construction of parks are related to residents’ walking behavior dynamically over time.

Furthermore, the estimated daily step count map was able to capture the significant decrease in step counts in the suburbs during heavy rains due to the typhoon. As with the neighborhood environment, natural disasters and infectious disease outbreaks can also contribute to lower physical activity levels [16, 17, 45]. A previous study indicated that proximity to parks can mitigate decreased walking during the COVID-19 pandemic [46], and assessing neighborhood resilience to disasters is an important topic in physical activity research. The approach of this study, which allows mobility observation on a retrospective time scale and over a wide geographic space by using large-scale GPS data, will be useful for an ex-post analysis of physical activity changes during disasters, the locations and timing of which are unpredictable.

This study has several limitations. The first is the bias of user attributes due to data collection methods. As the GPS logs and daily walking step data used were not sampled randomly but rather taken from users with specific applications installed, the generalizability of the results is limited. Especially, because the WalkCoin users were mostly in their 20–40 s, the behavioral characteristics of older people may be under-represented. Similarly, the age distribution of the GPS data sample used for the step count estimation may be biased towards middle-aged or younger people. In fact, the mean number of daily steps estimated by our model was higher than that reported by the walking volume survey conducted by the Miyagi Prefectural Government, which included the older population as described in the Results. However, the large-scale GPS-based walking observation is effective in capturing spatial and temporal characteristics of people’s walking behavior, such as the mitigation of a decrease in residents’ walking volume around suburban train stations or the clear decrease in step count in suburban areas during heavy rainfall. Second, there is a limitation regarding the GPS accuracy. As the GPS logs’ second-by-second time information was removed by Agoop before the data were provided to us and the logging intervals were irregular, it was difficult to identify detailed movements and stays at specific locations. To improve the accuracy of the model, only walking trips should be extracted from the GPS data acquired in more detailed time units; however, to our knowledge, data storing GPS logs and step counts with finer time units are not generally available in Japan. Furthermore, the GPS logs of timing of visits to underground spaces and buildings may have been excluded from the analysis due to decreased GPS positional accuracy. Future studies should build an application capable of obtaining second-by-second GPS logs, step counts, and questionnaire-based transportation mode, with place of visit needed to model the relationship between more realistic environmental exposures and step counts during walking. Third, this study used eight simple land use types as the indicators of environmental exposure; however, to further improve the model accuracy, other environmental factors related to walking, such as the condition of sidewalks, landscape, and safety, should be added. Finally, although the approach of this study is limited to linking the GPS-based environmental exposure and daily step counts, estimating the activity intensity during the trips is also needed for future application to physical activity research.

## Conclusion

This study built a model to estimate daily step counts from the movement trajectories using GPS logs and daily step count data obtained from the applications widely distributed to smartphone users in Japan. The relationship between environmental exposure and daily step counts estimated by the model is largely consistent with previous findings, indicating that observing the amount of walking with large-scale GPS data may provide a reasonable and reliable estimate of the amount of walking engaged in by people moving outside home. The methodology of this study, which utilizes continuously acquired large-scale GPS data, will allow for ex-post evaluation of the impact of environmental, social, and individual changes on walking conditions caused by policy interventions and unanticipated events, contributing to resilient and sustainable urban planning and public health measures for an increase in physical activity.

## Data Availability

GPS data used are available for purchase from Agoop Corp. and scripts for data processing and analysis are available from the corresponding author upon reasonable request.

## Abbreviations

GPS: Global positioning systems
COVID-19: Coronavirus disease 2019
UUID: Universally unique identifier
GIS: Geographic information system
